# A Social Capital Perspective on Social-Medical Collaboration in Community End-of-Life Care in Hong Kong

**DOI:** 10.1177/00302228211066678

**Published:** 2022-01-06

**Authors:** Wing-sun Chan, Laura Funk, Genevieve Thompson

**Affiliations:** 1 Department of Sociology and Criminology, 8664 University of Manitoba, Winnipeg, MB, Canada; 2 College of Nursing, 8664 University of Manitoba, Winnipeg, MB, Canada

**Keywords:** end-of-life care, social-medical collaboration, social capital, health promotion, interprofessional collaboration

## Abstract

Recent developments in Hong Kong end-of-life (EOL) care have shifted some caring work for dying people and their families to cross-disciplinary collaboration in community settings. Social-medical collaboration becomes especially important. This study aims to use social capital as an analytical lens to examine the processes and mechanisms of social-medical collaboration in EOL care and elucidate practice implications for engaging in the care of dying people and their families. Qualitative data were collected using in-depth interviews. Three major conceptual categories were generated through grounded theory methodology. They are (a) establishing trust through keeping clear and simple boundaries, (b) cultivating mutuality in the multi-disciplinary meeting, and (c) fostering social-medical collaboration in EOL care. Each new stage is based on the social capital accumulated in the previous one through the social interactions between professionals. Such theorization also provides insights into how to achieve effective social-medical collaboration in this context.

## Introduction

End-of-life (EOL) care is a significant public health issue in nations with aging populations. In general, the term EOL care encompasses support to physical, psychological, social, and spiritual dimensions for people approaching the EOL in the next 12 months (World Palliative Care Alliance, 2021). Like many developed industrial societies, the Hong Kong healthcare system aims to improve the quality of death for dying persons (Hospital Authority, 2017). Compared with other developed countries, the Hong Kong health service for dying persons is developed slower. According to the Quality of Death Index, Hong Kong was ranked as 22nd in the world, while Taiwan as the 6th, the US was ranked as 9th, Canada as the 11th, and Singapore as the 12th (The Economist Intelligence Unit, 2015).

One recent development in this regard involves engaging the social service sector to provide non-medical intervention to more fully achieve holistic EOL care (Hospital Authority, 2017). Standard care for dying people in Hong Kong appears to be grounded in a medicalized palliative care approach, including specialist-led services, largely hospital-based care, and a higher focus on cancer patients (Lam, 2019). Thus, the term "EOL care," in the Hong Kong context, indicates a distinct departure from more medicalized forms of palliative care towards more socially oriented care at the community level (Chan & Chow, 2019).

Although social-medical collaboration is often an essential feature of palliative, hospice, and EOL care development, interdisciplinary collaboration becomes increasingly important in the public health movement around EOL care (Karapliagou & Kellehear, 2018). Partnership at different levels in the system facilitates a care orientation that extends beyond disease management towards promoting health and well-being near the EOL (Karapliagou & Kellehear, 2018). However, the theorization of social processes in the interprofessional collaboration of EOL care is still underdeveloped in the discussion. Interprofessional collaboration is becoming increasingly common in primary healthcare practice and has been indicated as improving both the quality of EOL care and the stress of clinicians (Fox et al., 2019; Rechel, 2020; Rhee et al., 2020). Literature highlights barriers to effective EOL interprofessional collaboration, including how the varied professionals handle values, roles, communication, and coordination in their relationship (Dalkin et al., 2018; McDarby & Carpenter, 2019). More importantly, the literature also pinpoints the power imbalance between professionals as a critical barrier, which needs to be understood (Pype et al., 2018). As such, the social process of social-medical collaboration in community EOL care can provide insights on how to facilitate collaboration and reduce barriers to interprofessional collaborative EOL care in a hierarchical relationship (Ho et al., 2016; King et al., 2017).

Principally, social-medical collaboration can be characterized as an interpersonal process to achieve goals that cannot be reached only by an individual professional acting on their own (Bronstein, 2003). The nature of social-medical collaboration as an interpersonal process implicates dynamics and mechanisms of relationship development in a team-based setting (Cleary et al., 2019). It is essential to understand how to enable social-medical relationship development, thereby strengthening the quality of EOL care (Thiel et al., 2020).

Social capital is a useful analytical lens to explore the relational processes and mechanisms involved in social-medical collaboration (Lin, 2001). Although the concept of social capital has often been used in health-promotion research in palliative care, its application to the analysis of social-medical collaboration in the care of dying persons is still underdeveloped (Dempers & Gott, 2017; Rosenberg et al., 2015). Defined as “resources embedded in a social structure which are accessed and mobilized in purposive actions”, the capitalization process involved in social capital entails three aspects: structure, accessibility, and mobilization (Lin, 2001, p. 29). Although the concept of social capital can generate knowledge to improve the quality of health-promoting palliative care, more research exploring the concept in this field is needed.

Health-promoting palliative care was developed to emphasize the need for a joint effort between communities, governments, state institutions, and social or medical care organizations to enhance the wellness of persons facing life-threatening illnesses (Kellehear, 1999). The related concept of "compassionate communities", in particular, highlights how collaborative social relationships can strengthen community EOL care through enhancing the social atmosphere and institutional environment for dying persons and their caregiver(s) (Abbey et al., 2020). Although current discussions of social-medical collaboration tend to focus on the determinants of good quality interdisciplinary collaboration, such as communication, roles, knowledge, attitudes, and skills, there has been less attention to the relational processes and mechanisms involved in building higher-level collaboration in EOL care (Dudley et al., 2019; Otte et al., 2016).

As such, this study aims to use social capital as an analytical lens to examine the complex processes and mechanisms of social-medical collaboration in EOL care from practitioners' perspectives in two community-based social service agencies providing EOL care in Hong Kong communities. We also theorize those processes and mechanisms based on the sorting and analysis of substantial and theoretical codes accompanied by memo writing (Glaser & Strauss, 1967). As addressed in the discussion, attention to these complexities can enhance health promotion and community engagement in EOL care.

## Methods

This study aims to examine relational processes and mechanisms involved in developing social-medical collaboration in EOL care. Qualitative data were collected from practitioners in two community-based social service agencies providing EOL care in Hong Kong. Grounded Theory Methodology (GTM) was employed to allow the analysis to remain responsive to the data and facilitate inductive theorization while retaining some deductive elements about social relationships (Glaser & Strauss, 1967).

### Participant Recruitment, Sampling, and Data Collection

In-person, semi-structured, and in-depth qualitative interviewing was employed in this study. Data collection commenced in 2019 after obtaining approval from the Research Ethics Board at the university (Protocol #P2019: 045 (HS22756) approved on 23 April 2019). Participants were recruited between June 2019 and December 2019 based on their involvement with one of two service agencies: PARACLETE and the Life Rainbow project. PARACLETE (including "Hospice at Home") is affiliated with the Hospice and Bereavement Service, Hong Kong S.K.H Holy Carpenter Church District Elderly Community Centre. PARACLETE is the first project (since 2004) in Hong Kong to provide community support for people near the end of life and in bereavement ("Hospice at Home" started in 2015). The users are from the social service agencies in all districts in Hong Kong. The "Life Rainbow" project (started in 2015) is part of the Hong Kong Society of Rehabilitation, which has rich experience of social-medical collaboration because of its organizational background. The users are mainly from Hong Kong Island.

**Figure 1.**
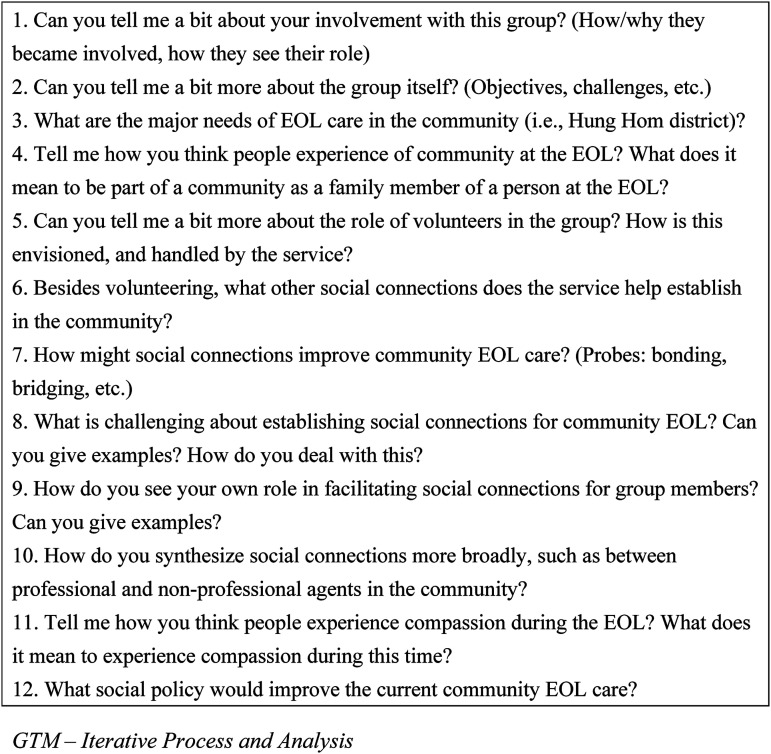
Interview questions for social workers and nurses

The first author recruited practitioners (social care organizers) and volunteers from these two organizations (*n* = 14). For the present analysis, only data from the practitioners are included here (*n* = 8) (see Table 1). In keeping with GTM, theoretical sampling was used, and involved the purposive recruitment of informants to provide further explanation for the developing conceptualization and theorization (Glaser & Strauss, 1967). The first author did all the individual interviews in fluent Cantonese and translated the verbatim Chinese transcripts into English (see Figure 1 for interview questions). Although the sampling strategy also sought to include medical professionals, the local researcher (first author) could not establish rapport with the Hong Kong Hospital Authority. Apart from the practitioners' viewpoints, this study could not explore the first-person views of medical professionals about the collaboration experience.

**Table 1.** Summary of Social Agency Practitioners Interviewed in PARACLETE and the Life Rainbow Community End-of-life (EOL) Care Projects.

Participant #	Role in Community EOL Care	Gender	Years Providing or Receiving Service
S001	Social worker (PARACLETE)	M	5
S002	Social worker (PARACLETE)	F	5
S003	Social worker (PARACLETE)	F	0.5
S004	Social worker (PARACLETE)	M	10
S005	Social worker (PARACLETE “Hospice at home”)	F	4
S006	Social worker (PARACLETE “Hospice at home”)	M	0.5
S010	Social worker (life rainbow)	F	3
S011	Nurse (life rainbow)	F	3
S012	Volunteer (life rainbow)	F	3

### GTM—Iterative Process and Analysis

Theoretical sampling was used to confirm or falsify the initial ideas in the earlier study stage (Strübing, 2007). After the first round of interviews with the organizers in PARACLETE (4 June 2019 to 26 July 2019), the first author developed coding around trust establishment (a finding which repeatedly appeared in the first round of interviews). The analytical thread of social capital in the collaborative relationship with the medical professionals was used to guide theoretical sampling with the organizers of Life Rainbow (29 August 2019 to 23 December 2019). The iterative-cyclical inquiry was used to discover the core categories alongside the consideration of additional evidence and arguments as the theory was developed (Strübing, 2007). The analysis process used the fundamental components of GTM—coding, developing categories, memo writing, theoretical sampling, constant comparative method, theoretical coding towards theoretical saturation. The preliminary analysis included open coding, which summarized “emerged” categories to inform the theoretical coding, followed by theoretical coding, which sharpened the research questions towards theoretical saturation (Glaser & Strauss, 1967). At saturation, new collected data did not offer additional insights for the conceptualization of the developing three stage theory of social-medical collaboration. Throughout the data analysis process, the first author regularly engaged in memo writing was before and after every interview. The goal was to clarify the association between concepts to assist with theoretical sorting (Charmaz, 2014). The emergent three-stage model of social-medical collaboration in community EOL care is thus based on a substantial number (i.e., 30-page) of memos as well as the interview data.

## Results

This in-depth analysis of the interview accounts of social agency practitioners in two community-based social service agencies providing community EOL care illustrates social-medical collaboration as an interpersonal process between practitioners in both disciplines. The social processes presented below include pre-conditions to and aspects of accessing and mobilizing social capital to formalize the social-medical relationship (Karam et al., 2018; Lin, 2001). Three stages of social-medical relationship development were theorized. Practitioners establish trust by keeping clear and simple boundaries, cultivating mutuality in the multi-disciplinary meeting, and fostering social-medical collaboration in EOL care. Each new stage is based on the social capital accumulated in the previous one. The accumulation of social capital hinges on the processes and mechanisms involved in the social interactions described below.

### Establishing Trust Through Keeping Clear and Simple Boundaries

The two social agencies regularly coordinate between medical professionals and the community of service users. These agencies need to establish trust with medical professionals (e.g., doctors and nurses in palliative wards and geriatric units). Medical professionals generally welcome the introduction of the psychosocial interventions associated with community-based EOL care. Still, they may be concerned about obligations and liabilities since they must adhere to hospital authority’s strict administrative and legal protocols. Therefore, social agency workers in this study referred to the need to be careful in the initial period of collaboration and strive to work out an exact position and roles; workers from these agencies seek to establish trust by keeping the relationship “clear and simple.”

On closer examination, however, “trust” appears to be more complex or problematic than might be assumed; it is not always “clear and simple.” For example, a social worker said that her experience of engagement with the hospital involves a person (the professor) knowing the physicians to help explain the intention of collaboration. Also, she let the medical professionals carefully consider any overlap in their labor division in the hospital. She also wanted to avoid any “ambiguity” in the trust establishing process because she realized the medical professionals could withdraw the engagement anytime without consequence.

With regards to establishing trust, at the very beginning, a university professor [who is the director of a community EOL care project in the university who takes care of the impact assessment of our project] helped us align with the senior management of the hospital [in the West New Territories]. We met many physicians of the oncology department, palliative ward, hospice centre, and geriatric department. … we worked with the palliative ward and found that the potential is highest [here]. Their primary concern is the role of the nurse in both parties if there is any overlap. (s5)

Agency employees spoke of carefully respecting the authority of the medical professional in their working relationships. For instance, they avoided making excessive recommendations for matters beyond their scope of service or asking irrelevant questions of medical professionals. In this regard, however, the concern is less about establishing trust than about fear of disrupting the current power imbalance (and incurring antipathy from the medical professionals, which would damage the working relationship). A nurse in “Life Rainbow” expressed her practice wisdom for establishing trust with medical professionals:

For the essence of cross-disciplinary collaboration [in the community EOL care], I still think mutual respect is the most crucial factor. I know the boundary in the collaborative relationship with the Hong Kong Hospital Authority. I know what I can do and what I must not. I also clearly know how to seek help appropriately from the right department. All in all, communication is the foundation in [cross-disciplinary] collaboration. (s11)

This participant emphasized mutual respect as an element of success in cross-disciplinary cooperation, and her talk belies a more uni-dimensional than the mutual application of “respect.” In this way, the implicit meaning of “trust” in relationships with medical professionals emerges as more about the latter's trust in social agency professionals not to overstep their boundaries. The "respect" reflects the power imbalance in these relationships, in contrast to providers' relationships with clients and families.

### Cultivating mutuality in the multi-disciplinary meeting

Social and medical professionals may prioritize distinctly specific considerations or concerns in relation to organizational issues of liability, administrative support, and risk management. In this section, social-medical mutuality refers to a relational status in which social and medical service professionals respond to each other on a similar or equal level of interdependence to leverage further collaboration—cultivating a sense of mutuality between medical and social professionals, just as in an interpersonal relationship, entails some performative strategies by social care professionals (Brown, 2016). Most notably, “ingratiation behaviors” enhance the potential for actors of different statuses to attribute likeability to the other (Jones & Pittman, 1982).

The occasion or opportunity for cultivating mutuality among medical and social professionals involved in community EOL care is the multi-disciplinary case meeting. This meeting plays an essential role through the mutual exchange of information (Arber, 2008). This meeting is a regular and formal occasion in which physicians, nurses, and social workers discuss the progress of community EOL care cases, identify professional needs (i.e., case handling), and how to coordinate to address client (and family) needs jointly.

Stimulated by regular case meetings and ongoing collaborative action on cases, different professionals can appreciate their own mutually supportive roles and functions (even if the physician might take the lead in some cases). This self-enhancing communication helps reduce power discrepancy between professionals by increasing interdependence (Jones & Pittman, 1982). However, social agency practitioners still face a distinct power imbalance in relationships with medical professionals. In response, social professionals might emphasize more performative aspects of mutuality. For example, they emphasize collaboration to nurture a sense of mutuality in the other through "opinion conformity" (Jones & Pittman, 1982). A social worker in "Hospice at Home" shared their experience of coordination during a case meeting:

We respect the physicians' viewpoints very much. We also tried to align our voices in the regular meeting. If we know the physicians use a view, we will keep consistent with their viewpoint afterwards. (s5)

This excerpt indicates how social workers actively work to achieve a sense of mutuality through opinion conformity, albeit fundamentally hierarchical (Jones & Pittman, 1982).

### Fostering Social-Medical Collaboration In End-Of-Life Care

Collaboration for community EOL care represents a closer, more thorough interdisciplinary exchange and coordination between workers to establish a smooth workflow, work norms, and finish tasks, and is based on and develops from a sense of trust and mutuality. This high-level social relationship development includes linking up care resources in communities, establishing ways of working together and mobilizing joint effort towards a specific operational goal.

A social worker from "Hospice at Home" told the focus of social-medical collaboration.

"Hospice at Home" has two working focuses. The first one is the collaboration at a hospital. We [the social workers and the medical professionals] thought how to coordinate our [social and medical] services to provide timely client support. The second focus is taking care of the emotional needs of the dying persons and their families [which is not the focus of service from the hospital]. (s6)

Through developed social connections and expanded networks, collaborative teams across medical and social sectors can better understand different care-related resources in communities.

Fostering collaborative community EOL care also involves creating ways of working together between social and medical service practitioners to virtually strengthen both social and medical service by a joint effort. A social worker in “Hospice at Home” described the community nurse's expectation about addressing the emotional needs of a client's husband when the client was readmitted to the hospital:

Sometimes, the community nurse knows that a client [that we both know] came to the hospital. And the condition of the client was getting worse. Since the hospital nurse had rarely met the client's husband, this nurse expected us to help find him and provide more support for him, especially emotional and bereavement support. We had often encountered this client's husband in the in-home visit. That's why I feel there is a robust collaborative relationship between the hospital and our project in case handling. (s5)

This excerpt revealed a set of work norms that fosters collaborative community EOL care—working together to close a service gap in the existing healthcare system. Collaboration between these sectors can reduce "medical dominance" and encourage forms of more "constructive medicalization" in EOL care (Broom & Woodward, 1996).

In 2015, the Hong Kong Hospital Authority updated its guidelines on Life-Sustaining Treatment in terminally ill persons, recommending that Advance Care Planning (ACP) be initiated in several situations with the consent of the dying person (Hospital Authority, 2015). Quality communication is a prerequisite to ACP discussions. However, medical professionals' training, skillset, and time demands make it challenging to initiate and have these discussions with dying persons and their family members. One of the functions of the project "Life Rainbow" is to assist with documenting clients' values and preferences for the ACP scheme. A social worker in "Life Rainbow" referred to the feedback they receive from physicians:

Recently, the physicians in Ruttonjee Hospital heard that we could help fill in the section of personal values in the ACP form. They [the physicians] know that this section is challenging to complete [because it is very time consuming and requires the helper to have high sensitivity]. Therefore, they hope we [the social workers] can help their team facilitate the patients to complete the whole section about psychosocial and personal value. (s10)

Although this organized example of medical-social collaboration is instrumentally driven by the need to complete the ACP form, it can benefit dying persons and their family members. The shared operational goal is to encourage clients and their family members actively to participate in EOL care decision-making.

## Discussion

This study introduces a novel social capital model of social-medical collaboration, attending to the complexities, nuances, and power dynamics involved in establishing trust, cultivating mutuality, and fostering collaborative community EOL care from the social agency practitioners' perspective. A social capital approach to developing relational, social-medical collaboration extends the practice of EOL care between disciplines to consider dynamics and strategies of social interactions rooted within distinctly hierarchical relationships (Firn et al., 2018). The emergent social-medical collaboration model in community EOL care includes the social processes and mechanisms of trust establishment, cultivating mutuality, and collaboration. It enriches the theorization of social capital development (i.e., pre-condition, capitalization, and outcomes) (Lin, 2001). Although data collection for the study faced some challenges due to political turmoil and the COVID-19 pandemic, the strength of this study lies in how it expands the application of social capital approach to theorize social-medical collaboration in EOL care with qualitative data. Such theorization provides insights on how to achieve effective social-medical collaboration in community EOL care. The three-stage theorization of social-medical collaboration has potential transferability to different contexts and can also prompt dialog about social-medical collaboration in EOL care, in discussions of the models’ fitness, understanding, generality, and control (Glaser & Strauss, 1967).

This study also provides practical recommendations to achieve better quality social-medical collaboration in community EOL care. Trust is a fundamental pre-condition of social capital development. When social and medical professionals join as a team to provide holistic support for EOL care cases, they recognize each other’s abilities (Firn et al., 2018). A key finding from this study was that keeping a clear and simple boundary is a crucial strategy to facilitate trust as a pre-condition of social capital development in interpersonal engagement. A carefully managed sense of a boundary, which avoids disrupting the current power imbalance, can reduce role misunderstandings, both from the practitioner and the managerial levels (Otte et al., 2016).

Mutuality cultivation is the most crucial stage of social capital development in social-medical collaboration. With trust established, interactants in the collaboration seek to move towards a more reciprocal relationship (Brown, 2016). A key finding from this study was that social professionals used a set of ingratiation behaviors to manage the impression of reciprocity (Jones & Pittman, 1982). In addition, mainly through multi-disciplinary case management meetings, a self-enhancing strategy reduces the power discrepancy in the relationship. Opinion conformity strategy maintains a harmonious reciprocal relationship. The practitioners continue and deepen the social exchange as a result (Brown, 2016).

At the highest level of social relationship development, participation in collaborative EOL care could transform the relationship into a formal one. Apart from resource coordination and navigation, collaboration appeared to be both simultaneously enhanced by, and to enhance, the implementation of the ACP scheme in this study. At this stage and particularly through the implementation of this scheme, the team-like relationship structure develops more specific shared goals, framework, and division of labor. The power imbalance between professionals could be reduced through a deeper mutual appreciation of strength and interdependence (Fernando & Hughes, 2019; Mertens et al., 2019).

In sum, theorizing with the aid of a social capital perspective provides insights into how to achieve an effective social-medical collaboration in community EOL care to strengthen health promotion and community engagement in the care of dying people and their families. In particular, social-medical collaboration needs to be acknowledged as fundamentally an inter-personal relationship (Pype et al., 2018). And the collaboration essentially needs the institutional support (Riotte et al., 2018).

Further, creating opportunities to establish trust is the first step of social-medical collaboration in community EOL care (Firn et al., 2018). Keeping a clear and simple boundary may be an effective way to start engagement, yet is not unproblematic, as it can place more onus on social care workers to defer to medical workers. With trust established, both medical and social practitioners may consider finding opportunities to access social capital and nurture mutuality by maintaining information exchange, effective communication, mutual learning, and division of labor.

Where practitioners realize opportunities for reciprocal relationships to transform into a higher-level collaboration, they may seek to formalize the relationship in a more tangible way to maintain the participation, communication, and knowledge sharing between both parties through the co-learning and co-design of interventions, such as through forming a community EOL care team, interdisciplinary case meeting, or partnership ACP program (Donesky et al., 2020; Riffin et al., 2016). As a final point, it should be noted that it is not always necessary to “force” a relationship up to the highest collaborative level without sufficient pre-conditions. Each stage can still contribute to positive EOL care outcomes because all the improvement of relationship can bring positive implications on the interdisciplinary collaboration and the quality of care (Sallnow & Paul, 2015). Yet, forcing the weak relationship (i.e., weak trust, communication, coordination, management, labor division, and leadership) in the collaboration to grow to a high-level collaboration may harm the quality of care (Oishi & Murtagh, 2014; Riotte et al., 2018). The capacity of collaboration could not fully address the psychological, social, and spiritual needs of the clients (Kellehear, 1999). For the key study limitation, due to the social unrest and outbreak of COVID-19, the first author could not engage with more agencies to explore more conceptual dimensions related to social-medical collaboration from the perspective of medical practitioners.

## Conclusion

The focus is on exploring the complex dynamics and mechanisms of relationships between social service agency workers and medical professionals, from the service agency perspective. Three stages of social-medical collaboration in community EOL care were delineated: establishing trust, cultivating mutuality, and fostering collaborative care. Future research should assess whether the general and particular aspects of the model, align with the perspectives of medical professionals working with community-based EOL social service agencies. More importantly, the transferability of the social capital model should be assessed in different healthcare and country context. Nevertheless, this study represents an important step towards continued research and theorization, providing comprehensive insights about fundamental concerns and challenges in the relational development of social-medical collaboration in community EOL care.
